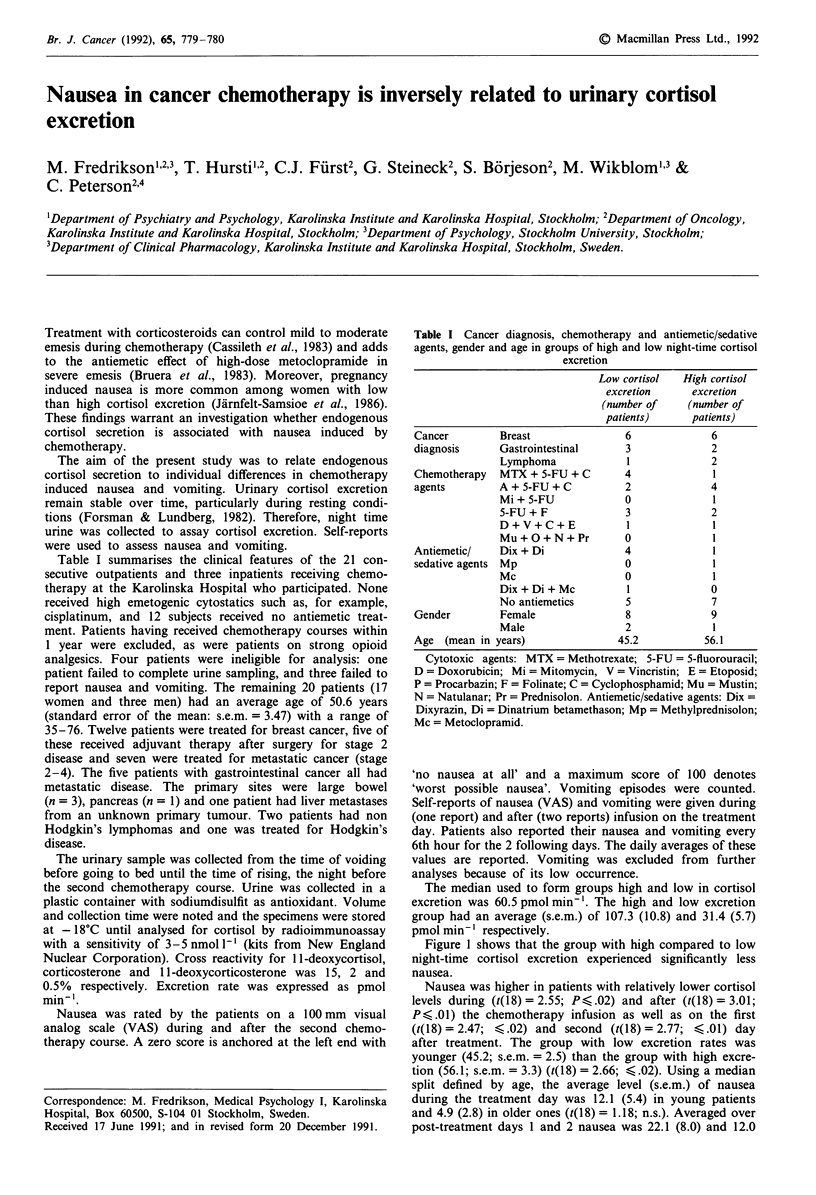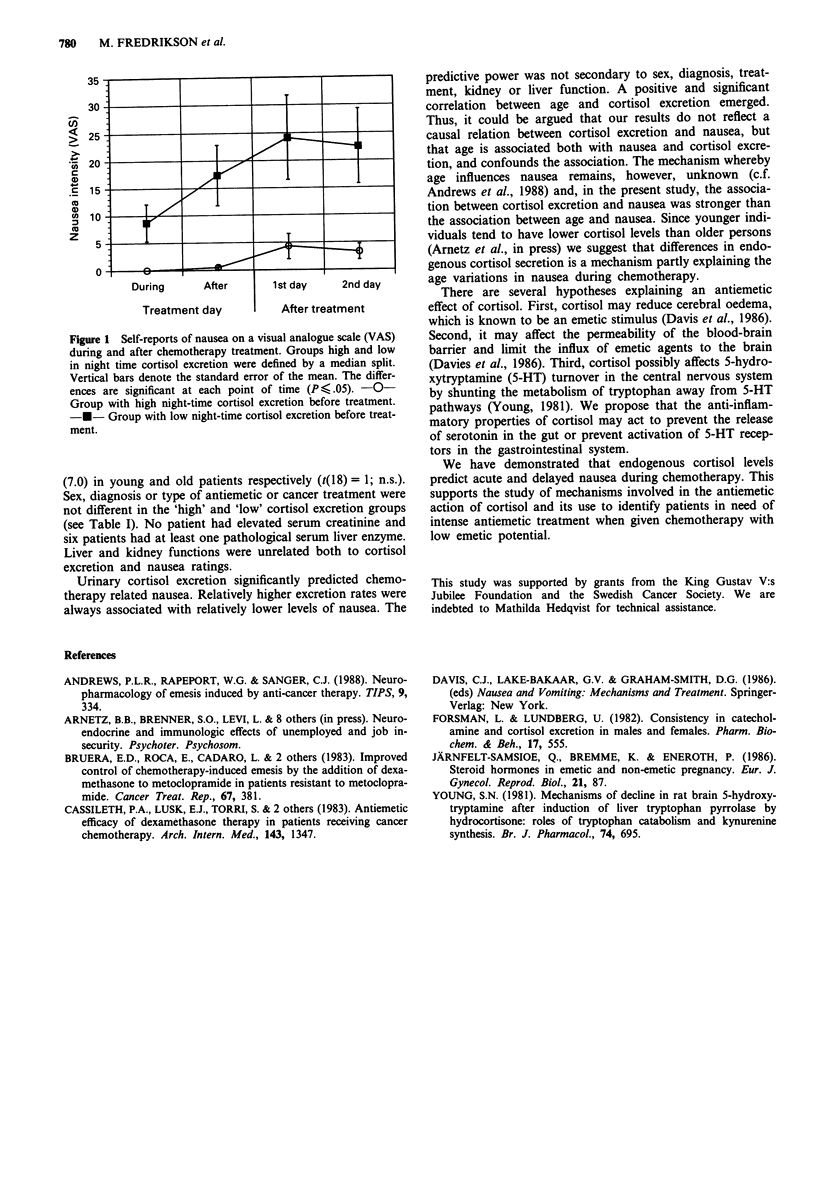# Nausea in cancer chemotherapy is inversely related to urinary cortisol excretion.

**DOI:** 10.1038/bjc.1992.165

**Published:** 1992-05

**Authors:** M. Fredrikson, T. Hursti, C. J. Fürst, G. Steineck, S. Börjeson, M. Wikblom, C. Peterson

**Affiliations:** Department of Psychiatry and Psychology, Karolinska Institute, Stockholm, Sweden.


Br. J. Cancer (1992), 65, 779–780 © Macmillan Press Ltd., 1992

### Nausea in cancer chemotherapy is inversely related to urinary cortisol excretion

M. Fredrikson[1,2,3], T. Hursti[1,2], C.J. Fürst[2], G. Steineck[2], S. Börjeson[2], M. Wikblom[1,3] & C. Peterson[2,4]

[1]*Department of Psychiatry and Psychology, Karolinska Institute and Karolinska Hospital, Stockholm;* [2]*Department of Oncology, Karolinska Institute and Karolinska Hospital, Stockholm;* [3]*Department of Psychology, Stockholm University, Stockholm;* [3]*Department of Clinical Pharmacology, Karolinska Institute and Karolinska Hospital, Stockholm, Sweden.*

Treatment with corticosteroids can control mild to moderate emesis during chemotherapy (Cassileth *et al.*, 1983) and adds to the antiemetic effect of high-dose metoclopramide in severe emesis (Bruera *et al.*, 1983). Moreover, pregnancy induced nausea is more common among women with low than high cortisol excretion (Järnfelt-Samsioe *et al.*, 1986). These findings warrant an investigation whether endogenous cortisol secretion is associated with nausea induced by chemotherapy.

The aim of the present study was to relate endogenous cortisol secretion to individual differences in chemotherapy induced nausea and vomiting. Urinary cortisol excretion remain stable over time, particularly during resting conditions (Forsman & Lundberg, 1982). Therefore, night time urine was collected to assay cortisol excretion. Self-reports were used to assess nausea and vomiting.

Table I summarises the clinical features of the 21 consecutive outpatients and three inpatients receiving chemotherapy at the Karolinska Hospital who participated. None received high emetogenic cytostatics such as, for example, cisplatinum, and 12 subjects received no antiemetic treatment. Patients having received chemotherapy courses within 1 year were excluded, as were patients on strong opioid analgesics. Four patients were ineligible for analysis: one patient failed to complete urine sampling, and three failed to report nausea and vomiting. The remaining 20 patients (17 women and three men) had an average age of 50.6 years (standard error of the mean: s.e.m. = 3.47) with a range of 35–76. Twelve patients were treated for breast cancer, five of these received adjuvant therapy after surgery for stage 2 disease and seven were treated for metastatic cancer (stage 2–4). The five patients with gastrointestinal cancer all had metastatic disease. The primary sites were large bowel ($n = 3$), pancreas ($n = 1$) and one patient had liver metastases from an unknown primary tumour. Two patients had non Hodgkin's lymphomas and one was treated for Hodgkin's disease.

The urinary sample was collected from the time of voiding before going to bed until the time of rising, the night before the second chemotherapy course. Urine was collected in a plastic container with sodiumdisulfit as antioxidant. Volume and collection time were noted and the specimens were stored at −18°C until analysed for cortisol by radioimmunoassay with a sensitivity of 3–5 nmol $l^{-1}$ (kits from New England Nuclear Corporation). Cross reactivity for 11-deoxycortisol, corticosterone and 11-deoxycorticosterone was 15, 2 and 0.5% respectively. Excretion rate was expressed as pmol $min^{-1}$.

Nausea was rated by the patients on a 100 mm visual analog scale (VAS) during and after the second chemotherapy course. A zero score is anchored at the left end with

Correspondence: M. Fredrikson, Medical Psychology I, Karolinska Hospital, Box 60500, S-104 01 Stockholm, Sweden.
Received 17 June 1991; and in revised form 20 December 1991.

**Table I** Cancer diagnosis, chemotherapy and antiemetic/sedative agents, gender and age in groups of high and low night-time cortisol excretion

| | | *Low cortisol excretion (number of patients)* | *High cortisol excretion (number of patients)* |
|---|---|---|---|
| Cancer diagnosis | Breast | 6 | 6 |
| | Gastrointestinal | 3 | 2 |
| | Lymphoma | 1 | 2 |
| Chemotherapy agents | MTX + 5-FU + C | 4 | 1 |
| | A + 5-FU + C | 2 | 4 |
| | Mi + 5-FU | 0 | 1 |
| | 5-FU + F | 3 | 2 |
| | D + V + C + E | 1 | 1 |
| | Mu + O + N + Pr | 0 | 1 |
| Antiemetic/ sedative agents | Dix + Di | 4 | 1 |
| | Mp | 0 | 1 |
| | Mc | 0 | 1 |
| | Dix + Di + Mc | 1 | 0 |
| | No antiemetics | 5 | 7 |
| Gender | Female | 8 | 9 |
| | Male | 2 | 1 |
| Age (mean in years) | | 45.2 | 56.1 |

Cytotoxic agents: MTX = Methotrexate; 5-FU = 5-fluorouracil; D = Doxorubicin; Mi = Mitomycin, V = Vincristin; E = Etoposid; P = Procarbazin; F = Folinate; C = Cyclophosphamid; Mu = Mustin; N = Natulanar; Pr = Prednisolon. Antiemetic/sedative agents: Dix = Dixyrazin, Di = Dinatrium betamethason; Mp = Methylprednisolon; Mc = Metoclopramid.

'no nausea at all' and a maximum score of 100 denotes 'worst possible nausea'. Vomiting episodes were counted. Self-reports of nausea (VAS) and vomiting were given during (one report) and after (two reports) infusion on the treatment day. Patients also reported their nausea and vomiting every 6th hour for the 2 following days. The daily averages of these values are reported. Vomiting was excluded from further analyses because of its low occurrence.

The median used to form groups high and low in cortisol excretion was 60.5 pmol $min^{-1}$. The high and low excretion group had an average (s.e.m.) of 107.3 (10.8) and 31.4 (5.7) pmol $min^{-1}$ respectively.

Figure 1 shows that the group with high compared to low night-time cortisol excretion experienced significantly less nausea.

Nausea was higher in patients with relatively lower cortisol levels during ($t(18) = 2.55$; $P \leqslant .02$) and after ($t(18) = 3.01$; $P \leqslant .01$) the chemotherapy infusion as well as on the first ($t(18) = 2.47$; $\leqslant .02$) and second ($t(18) = 2.77$; $\leqslant .01$) day after treatment. The group with low excretion rates was younger (45.2; s.e.m. = 2.5) than the group with high excretion (56.1; s.e.m. = 3.3) ($t(18) = 2.66$; $\leqslant .02$). Using a median split defined by age, the average level (s.e.m.) of nausea during the treatment day was 12.1 (5.4) in young patients and 4.9 (2.8) in older ones ($t(18) = 1.18$; n.s.). Averaged over post-treatment days 1 and 2 nausea was 22.1 (8.0) and 12.0

**Figure 1** Self-reports of nausea on a visual analogue scale (VAS) during and after chemotherapy treatment. Groups high and low in night time cortisol excretion were defined by a median split. Vertical bars denote the standard error of the mean. The differences are significant at each point of time ($P \leqslant .05$). —O— Group with high night-time cortisol excretion before treatment. —■— Group with low night-time cortisol excretion before treatment.

(7.0) in young and old patients respectively ($t(18) = 1$; n.s.). Sex, diagnosis or type of antiemetic or cancer treatment were not different in the 'high' and 'low' cortisol excretion groups (see Table I). No patient had elevated serum creatinine and six patients had at least one pathological serum liver enzyme. Liver and kidney functions were unrelated both to cortisol excretion and nausea ratings.

Urinary cortisol excretion significantly predicted chemotherapy related nausea. Relatively higher excretion rates were always associated with relatively lower levels of nausea. The predictive power was not secondary to sex, diagnosis, treatment, kidney or liver function. A positive and significant correlation between age and cortisol excretion emerged. Thus, it could be argued that our results do not reflect a causal relation between cortisol excretion and nausea, but that age is associated both with nausea and cortisol excretion, and confounds the association. The mechanism whereby age influences nausea remains, however, unknown (c.f. Andrews *et al.*, 1988) and, in the present study, the association between cortisol excretion and nausea was stronger than the association between age and nausea. Since younger individuals tend to have lower cortisol levels than older persons (Arnetz *et al.*, in press) we suggest that differences in endogenous cortisol secretion is a mechanism partly explaining the age variations in nausea during chemotherapy.

There are several hypotheses explaining an antiemetic effect of cortisol. First, cortisol may reduce cerebral oedema, which is known to be an emetic stimulus (Davis *et al.*, 1986). Second, it may affect the permeability of the blood-brain barrier and limit the influx of emetic agents to the brain (Davies *et al.*, 1986). Third, cortisol possibly affects 5-hydroxytryptamine (5-HT) turnover in the central nervous system by shunting the metabolism of tryptophan away from 5-HT pathways (Young, 1981). We propose that the anti-inflammatory properties of cortisol may act to prevent the release of serotonin in the gut or prevent activation of 5-HT receptors in the gastrointestinal system.

We have demonstrated that endogenous cortisol levels predict acute and delayed nausea during chemotherapy. This supports the study of mechanisms involved in the antiemetic action of cortisol and its use to identify patients in need of intense antiemetic treatment when given chemotherapy with low emetic potential.

This study was supported by grants from the King Gustav V:s Jubilee Foundation and the Swedish Cancer Society. We are indebted to Mathilda Hedqvist for technical assistance.

### References

ANDREWS, P.L.R., RAPEPORT, W.G. & SANGER, C.J. (1988). Neuropharmacology of emesis induced by anti-cancer therapy. *TIPS*, **9**, 334.

ARNETZ, B.B., BRENNER, S.O., LEVI, L. & 8 others (in press). Neuroendocrine and immunologic effects of unemployed and job insecurity. *Psychoter. Psychosom.*

BRUERA, E.D., ROCA, E., CADARO, L. & 2 others (1983). Improved control of chemotherapy-induced emesis by the addition of dexamethasone to metoclopramide in patients resistant to metoclopramide. *Cancer Treat. Rep.*, **67**, 381.

CASSILETH, P.A., LUSK, E.J., TORRI, S. & 2 others (1983). Antiemetic efficacy of dexamethasone therapy in patients receiving cancer chemotherapy. *Arch. Intern. Med.*, **143**, 1347.

DAVIS, C.J., LAKE-BAKAAR, G.V. & GRAHAM-SMITH, D.G. (1986). (eds) *Nausea and Vomiting: Mechanisms and Treatment*. Springer-Verlag: New York.

FORSMAN, L. & LUNDBERG, U. (1982). Consistency in catecholamine and cortisol excretion in males and females. *Pharm. Biochem. & Beh.*, **17**, 555.

JÄRNFELT-SAMSIOE, Q., BREMME, K. & ENEROTH, P. (1986). Steroid hormones in emetic and non-emetic pregnancy. *Eur. J. Gynecol. Reprod. Biol.*, **21**, 87.

YOUNG, S.N. (1981). Mechanisms of decline in rat brain 5-hydroxytryptamine after induction of liver tryptophan pyrrolase by hydrocortisone: roles of tryptophan catabolism and kynurenine synthesis. *Br. J. Pharmacol.*, **74**, 695.